# Willingness to pay for a cure of low-risk melanoma patients in Germany

**DOI:** 10.1371/journal.pone.0197780

**Published:** 2018-05-24

**Authors:** Matthias Augustin, Christine Blome, Andrea Forschner, Ralf Gutzmer, Axel Hauschild, Lucie Heinzerling, Elisabeth Livingstone, Carmen Loquai, Dirk Schadendorf, Jochen Utikal, Tobias Wagner, Sophia Wilden, Katharina C. Kähler

**Affiliations:** 1 Institute for Health Services Research in Dermatology and Nursing (IVDP), University Medical Center Hamburg, Germany; 2 Department of Dermatology, Eberhard-Karls University of Tübingen, Tübingen, Germany; 3 Skin Cancer Center Hannover, Department of Dermatology, Hannover Medical School, Hannover, Germany; 4 Skin Cancer Center, Department of Dermatology, University Hospital Schleswig-Holstein (UKSH), Campus Kiel, Kiel, Germany; 5 Department of Dermatology, University Hospital Erlangen, Erlangen, Germany; 6 Department of Dermatology, University Hospital Essen, University Duisburg-Essen, Essen, Germany; 7 Department of Dermatology, University of Mainz, Mainz, Germany; 8 Skin Cancer Unit, German Cancer Research Center (DKFZ), Heidelberg, Germany; 9 Department of Dermatology, Venereology and Allergology, University Medical Center Mannheim, Mannheim, Germany; University of Queensland Diamantina Institute, AUSTRALIA

## Abstract

Malignant melanoma is potentially life-threatening but in most cases curable if detected early. Willingness to pay (WTP) is a preference-based construct that reflects burden of disease by assessment of the monetary value for a hypothetical cure from disease. Since WTP (directly as total amount of money) has not been assessed so far in patients with low risk melanoma, it was interesting to gain insights in this patient population and then, in a second step, compare it directly with the WTP of their treating dermato-oncologists. WTP was assessed in 125 patients with low-risk melanoma and additionally in 105 treating physicians, asking for the one-time and continuous payments they would be willing to make for a sustainable cure, both as absolute sums and as percentages of monthly income. The median WTP based on one-time payment was €10,000 for patients and €100,000 for physicians; relative numbers were 100% versus 300% of monthly income. For continuous monthly payments, WTP was €500 for patients and €1000 for physicians, relative numbers 25% and 50% of income, respectively. Even after controlling for income differences, there was a significantly higher WTP in physicians for all four questions. Compared to patients with chronic skin diseases such as vitiligo, rosacea, atopic eczema and psoriasis, patients with low-risk melanoma showed a significantly higher WTP. Our data suggest that there is a relevant burden of disease even in patients with low-risk tumors. Higher WTP of physicians underlines the prevalence of differences in disease perception.

## Introduction

The incidence of cutaneous malignant melanoma has been increasing steadily for the last 50 years in fair-skinned populations and is one of the fastest growing cancer entities [1]. Melanoma is a potentially curable cancer, in patients with advanced disease prognosis remains poor [2]. For many patients, the diagnosis of melanoma is accompanied by considerable psychological, social and emotional consequences, disturbing QoL, self-esteem, the social roles and relationships and possibly the financial situation. It is well understood that age, gender and personality characteristics influence the burden felt by melanoma [3,4]. Approximately 30% of all patients diagnosed with melanoma report levels of psychological distress indicative of the need for clinical intervention [5].

The approval of new drugs such as immune checkpoint inhibitors and targeted therapies markedly improved overall survival [6]. However, this treatment benefit is associated with immune-related adverse events (irAEs) including colitis, hypophysitis and hepatitis potentially impairing QoL and with the need of a specific side effect management [7]. Thus, further analyses of the individual patient preference for the appropriate therapy are needed in order to gain more information on the ideal trade-off between efficacy and toxicity. Vast differences in therapy preferences as well as in WTP between patients and treating physicians have been shown [8]. The individual patient preference may also be driven by parameters like fear of recurrence and may be supported by the burden felt from the disease.

Willingness to pay (WTP) is a measure of disease burden and is defined as the amount of money subjects are willing to spend if it would cure them from disease associated with a specific health state. For a standardized assessment of WTP, hypothetical scenarios can be used [9, 10]. WTP has been measured in several dermatological diseases [11–16].

A study performed in the US, which included 150 cancer patients, showed that 25% of melanoma patients were willing to pay at least €32,865 ($45,000) choosing a therapy with a chance of a longer survival, but possibly not resulting in an improvement of QoL at all. Patients would be willing to pay an average of €26,515 ($36,305) for an additional year of survival and even €39,703 ($54,362) for a not determined time of longer survival [17]. Jenkins et al. [18] found that about 15% of cancer patients would be willing to pay about €4,860 (£4,000) per month for an anti-cancer drug of which only every fifth patient would benefit. Interestingly, even more healthy controls (20%) were willing to pay this sum in the hypothetical scenario of being diagnosed with cancer. Korean metastatic breast cancer patients were willing to pay an average of 7555 US$ per month to return to their pre-cancer health state. Recently diagnosed patients had the highest WTP (11,254 US$). WTP was associated with the patient's education level, income, Eastern Cooperative Oncology Group performance status, and their experience of lymphedema [19].

A study in German patients measured WTP and preference of quality versus length of life in small samples of melanoma patients, physicians and healthy controls. As an example for a high-priced drug with marginal benefit in many cases, the authors investigated the attitudes towards ipilimumab by a time trade-off between quality and length of life. The judgment about the benefits differed widely between the groups, with physicians being most critical towards therapy [8]. The German study also found a large difference between healthy volunteers and melanoma patient.

The discussion about the risk-benefit ratio of a cancer treatment is important since physicians rate it differently from patients. Therapy decisions have to be discussed with each patient on an individual level keeping in mind these differences.

Physicians are endued with knowledge and clinical experience with regard to therapies, progression of disease and prognosis. The process of clinical decision making is probably highly influenced by physicians’ impression of the patients’ needs, social circumstances and QoL. Very often physicians are influenced by disadvantageous disease outcomes and therefore might be willing to invest more against a disease.

Some physicians might also integrate their personal values. In contrast, others might explicitly avoid this and probably pronounce the chance of longer survival even if they would personally not favor the therapy. We therefore were not only interested in the patients’ WTP but also in the WTP of physicians treating melanoma patients.

To the best of our knowledge, this is the largest study assessing WTP in a very homogenous population of low-risk melanoma patients. Additionally, we compared patients’ to physicians’ WTP and as well as to WTP of patients with nonmalignant skin diseases. Further, attitudes and variables that influence the patients’ preferences were analyzed.

## Materials and methods

### Study design and centers

This cross-sectional cohort study was conducted in 10 skin cancer centers in Germany. Patient questionnaires as well as physician questionnaires with the same items concerning WTP were distributed in the centers. The study was approved by the ethics committee of the University of Kiel (vote number 142/12) and secondarily by regional all ethics committees of contributing centers before start of study. Written informed consent was obtained from patients and physicians.

### Participants

We recruited physicians working at the study melanoma centers, with familiarity with the diagnosis and treatment of malignant melanoma. The comparator group of low risk melanoma patient was chosen due to ethical reasons because we intended to have a cohort that knows the situation of a cancer diagnosis but no in the situation to choose a treatment in a real life setting. Physicians with daily contact with these patients were chosen for this trial.

The comparator group consisted of patients with low-risk melanoma after surgical resection [20]. Patients with a first diagnosis of melanoma <8 weeks before signature of informed consent were excluded to avoid ethical conflicts. Further, patients with planned elective or sentinel lymph node dissection, evidence of metastasis, severe co-morbidities or indication for adjuvant treatment were excluded. The criteria for low-risk melanoma were defined as patients with stage IA according to the revised melanoma AJCC staging system 2009 (pT1a tumor with a thickness of ≤1 mm and without ulceration).

### Questionnaires (S1 and S2 Figs)

#### Socio-demographics

Patients and physicians were asked to indicate socio-demographic data including gender, age, nationality, living situation, marital status and net income. The patient questionnaire also comprised questions on education, professional situation, employment, selected co-morbidities and regular drug intake. Physicians were further asked how long they had been working as a dermatologist and how frequently they treated patients with malignant melanoma.

#### HADS

The Hospital Anxiety and Depression Scale (HADS) is a self-assessment tool for detection of depression, anxiety and emotional distress. It consists of two subscales: The depression scale (HADS-D) and the anxiety scale (HADS-A) are calculated from seven items each. Patients are asked to rate the relevance of each question on a Likert scale from 0 (not/minimally present) to 3 (maximally present). Thus, the subscales range from 0 to 21 points [21]. Scores ranging from 0 to 7 points are considered to be within normal range; 8 to 10 points represent a borderline score. A score of 11 points or above indicates clinically relevant anxiety or depression. The HADS has been shown to be a valid and reliable assessment tool in somatically ill patients [22].

#### EQ-5D

The generic health status was examined using the second part of the EuroQol questionnaire (EQ-5D). The second part consists of a visual analogue scale (EQ VAS) with the endpoints labelled ‘best imaginable health state’ at the top and ‘worst imaginable health state’ at the bottom having numerical values of 100 and 0, respectively [23]. For our analysis, we used the EQ VAS.

### Assessment of willingness to pay

WTP was assessed as published previously [12, 15, 24, 25]. Patients and physicians were asked to state the absolute amount of money they would be willing to spend for complete cure from melanoma. Further, we asked for the percentage of their income patients were willing to pay. Both parameters were related to a one-time spending and to monthly payments for sustainable cure of disease.

### Statistics

Data were analyzed with IBM SPSS version 21 for Microsoft Windows. For all variables, descriptive statistics were computed. Mann-Whitney U-tests or Kruskal-Wallis tests were used to analyze the relationship between categorical and interval-scaled variables. Correlation analysis was performed with Spearman’s rank test. Two-tailed tests of significance were considered to be significant at a p-value <0.05 and highly significant at p <0.01. Distribution classes of WTP were defined visually by SPSS function for visual binning with a maximum of ten categories. For binary logistic regression, absolute one-time payment as the dependent variable–dichotomized by median split–was used. Analysis of covariance was carried out by ANCOVA. In course of significant Levene’s tests indicating unequal variances, post-hoc adjustment was performed by Tamhane-T2 post-test representing a combined multiple comparison and range test.

## Results

### Study cohort

A total of 157 out of 174 patient questionnaires and 116 out of 119 physician questionnaires were returned. In patients, the missing values for the absolute amounts of money (35.0% for one-time payment and 31.8% for continuous payment) were higher than for payment expressed as a percentage of income (24.1% for one-time payment and 26.8% for continuous payment). This difference was absent in physicians (25.9% missing answers in every category).

For analysis, we used all questionnaires in which a statement for at least two of the WTP questions of one category (absolute payment and relative payment) was given, thereby excluding 31 patients (19.7%) and 9 physicians (7.8%). We checked for subjects having indicated a higher monthly payment than one-time payment or who would spend a higher percentage of income monthly than as one-time payment to exclude obvious inconsistencies. Thereby, one patient and two physicians were excluded. Consequently, a total of 230 questionnaires (125 patient and 105 physician questionnaires) were analyzed.

### Sociodemographic characteristics

The mean age of patients was 54.5 (±12.6) years, the cohort was nearly equally split by gender (47.2% female, n = 59). 14.4% of patients (n = 18) lived alone. The median category of monthly income was between €1,600 and 2,400, 19.2% of patients (n = 24) did not give information about their income. 17.6% of patients (n = 22) had been confronted with cancer in their past, most of them with skin cancer. 3 out of 40 patients had formerly or currently a basal cell carcinoma. The rest of the patients suffered from cancers like secondary melanoma, uterus carcinoma, testicular cancer, lymphoma 75.2% of patients (n = 94) indicated that they have closely related persons affected by cancer (Table 1).

**Table 1:** Sociodemographic facts.

	Patients (n = 125)		Physicians (n = 105)	
*Mean age* in years (SD, range)	54.5 (±12.6, 25–88)		35.2 (±7.2, 25–62)	
	n	%	n	%
***Gender*** (female)	58	46.8	68	64.8
***Monthly income (Euro)***				
▪ <1,000	13	10.4	0	0.0
▪ 1,000–1,600	23	18.4	3	2.9
▪ 1,600–2,400	26	20.8	26	24.8
▪ 2,400–4,000	15	12.0	30	28.6
▪ 4,000–6,4000	17	13.6	15	14.3
▪ >6,400	7	5.6	4	3.8
▪ Missing	24	19.2	27	25.7
***Education level***				
▪ Low^1^	20	16.0	0	0.0
▪ Intermediate^2^	40	32.0	0	0.0
▪ High^3^	62	49.6	105	100.0
***Professional qualification***				
▪ University or polytechnic degree	46	36.8	105	100.0
▪ Apprenticeship	73	58.4	0	0.0
***Marital status***				
▪ Married/partnership	101	80.8	72	68.6
▪ Widowed	5	4.0	0	0.0
▪ Divorced/separated	9	7.2	1	1.0
▪ Single	9	7.2	29	27.6
***Living alone***	18	14.4	31	29.5
***Employment status***				
▪ Employed	79	63.2	105	100.0
▪ Not working^4^	46	26.8	0	0.0
***Other somatic disease***	32	25.6		*)
***Own malignancies in the past***	22	17.6	4	3.8
***Malignancies of closely related persons***	94	75.2	61	58.1

*) not assessed in physicians.

^1^less than certificate of intermediate secondary education.

^2^certificate of intermediate secondary education.

^3^polytechnical secondary school, advanced technical college entrance qualification or general qualification for university entrance.

^4^including Leave of absence, sabbatical, retiree, pensioner, early retirement, housewife/homemaker, student.

The mean age of physicians was 35.2 (± 7.2) years, about two thirds of physicians were female (64.8%, n = 68). The median monthly income was higher in physicians (median category €2,400–4,000). 29.5% of physicians (n = 31) lived alone, 25.7% of physicians (n = 27) did not give information about their income. Only 3.8% of physicians (n = 4) had been confronted with cancer in their past. 58.1% of physicians (n = 61) indicated that they have closely related persons affected by cancer (Table 1).

92.4% of physicians (n = 97) indicated that they regularly treated patients with melanoma. Within this group, each physician treated a median number of 40 patients monthly (range 1–400). Of these, 26.7% (n = 28) named a number above 100 patients monthly. On average, physicians had 7.1 years (±6.5 years, range 0–29) of professional experience in dermatology. One third (32.0%, n = 33) of physicians had been working for ≥10 years as a dermatologist.

### EQ-5D and HADS

The mean EQ-VAS was 83.8 (±14.7, range 20–100) in melanoma patients. Thus, a better subjective health could be assumed compared to 61.9 (±22.0) in WTP studies on atopic dermatitis [18], 62.0 (±21.0) in psoriasis [19], 67.3 (±20.6) in rosacea [9] and comparable to 83.6 in vitiligo [12]. 90.4% reported an EQ-VAS >50 and thus a relatively good subjective health. In physicians, a higher mean EQ-VAS of 92.8 was found (±7.0, range 68–100; p<0.001). 96.2% of physicians reported an EQ-VAS >50. Neither in the patient cohort nor in the physicians`cohort significant associations between EQ-VAS and WTP were detected.

HADS-A (mean 4.4 ±3.3, range 0–14) and HADS-D (mean 2.4 ±2.9, range 0–11) showed low average scores. A small group of 9 patients (7.2%) with borderline HADS-D and 2 patients (1.6%) with clinically relevant HADS-D were found. Conspicuous HADS-A scores were more frequent; 19 patients (15.2%) had borderline scores and 7 patients (5.6%) a clinically relevant score.

### Willingness to pay

As a one-time payment for sustainable cure from melanoma, patients were willing to pay €48,900 on average (± €148,400, range €200-€1,000,000) while physicians would spend €318,100 (± €1,607,800, range €500-€5,000,000). The medians of both groups differed 10-fold (€10,000 in patients, €100,000 in physicians). In contrast to one-time payment, median WTP for a continuous payment differed only slightly between patients (€1,932 ± €9,932, range €20,000-€100,000,000) and physicians (€2,076 ± €2,555; range €75,000-€100,000) and medians just 2-fold (€500 in patients, €1,100 in physicians).

One-time payment expressed as a percentage of monthly income showed high variation within both groups. Patients were willing to spend 3,108% (±227,611%, range 10–300,000) on average. Physicians, again, indicated much higher WTP (6,253% ± 33,029 on average, range 100–300,000). Continuous payment stated as percentage of monthly income revealed a lower median WTP in the patient cohort with 25% (range 3–4,000) compared to 50% (range 1–300) in physicians (), Distribution of willingness to pay).

There were high correlations between one-time and continuous payments stated as an absolute sum of money (r = 0.69, p<0.001 in patients and r = 0.59, p<0.001 in physicians, respectively). In WTP expressed as a percentage of income, we found a weaker correlation in patients (r = 0.31, p = 0.001) and no correlation in physicians (r = 0.10, p = 0.35).

### Comparison of patients’ and physicians’ WTP

Our analysis showed a clear contrast between patients’ and physicians’ WTP (Table 2), with significantly differing answers in all of the four questions indicating a higher WTP in physicians (p<0.001 for each question).

**Table 2:** Comparison of patients’ and physicians’ WTP.

Willingness to pay	Median		U test	
	Patients	Physicians	Z	p
one-time payment [k€]	10.0	100.0	-8.1	<0.001
one-time payment [% of income]	100.0	300.0	-4.1	<0.001
continuous payment [€]	500	1,000	-6.1	<0.001
continuous payment [% of income]	25.0	50.0	-3.9	<0.001

### Patient and physician characteristics associated with WTP

In patients, we found a strong positive correlation between WTP and income for absolute one-time payment (r = 0.41, p<0.001) and absolute continuous payment (r = 0.39, p<0.001). In physicians, this association was absent. For WTP expressed as a percentage of income, no associations between WTP and income could be found in both groups.

HADS was part of the patients’ questionnaire. Here, we found a significant (p<0.05) correlation of HADS-A with all WTP questions (r-range 0.17–0.24) while a correlation of WTP and HADS-D was absent. We found no systematic associations between WTP and subjective health state assessed by EQ-VAS. Higher age was a moderately negative predictor for one-time payment in patients (r = -0.19, p = 0.03 for absolute sum, r = -0.21, p = 0.01 for income values). Again, this association was absent in physicians.

Despite of lower median income in women, WTP was not significantly lower than in men; they even indicated descriptively higher median sums for one-time payment (in absolute as well as in relative values). Physicians living alone tended to show lower WTP in all categories with significant differences in absolute one-time payment (p = 0.02) and continuous payment related to income (p = 0.04) while patients did not show such differences.

For both questions concerning one-time payment (in € and % of income), higher education level was associated with higher WTP (r = 0.43, p<0.001 and r = 0.26, p = 0.002 respectively). In case of continuous payment (in € and % of income), this was only the case for the amount in € (r = 0.25, p = 0.005). Interestingly, monthly WTP expressed as a percentage of income did not vary by education level despite the fact that low education level is frequently associated with lower income. Employed patients showed a higher WTP for both questions concerning one-time payment (absolute and relative amount of money, p<0.05 each) but not for continuous monthly payment. Consistent with the fact that profession is a strong indicator of financial abilities, we found significantly higher WTP expressed in absolute sums in higher-educated patients.

In patients with a university degree, median WTP was approximately doubled for absolute one-time payment (€22500 versus €10000, p = 0.001) and absolute numbers of monthly payment (€600 versus €300, p = 0.002). Patients with at least one severe co-morbidity (n = 32, 25.6%) did not differ in WTP from patients without severe co-morbidity. Own experience with cancer of cancer in closely related persons also did not influence the WTP in either patients (n = 94) or physicians (n = 61).

### Multiple regression model and ANCOVA

For a multiple regression model for the prediction of the individual WTP (one-time payment in €), three variables were selected (age, gender, income) that were assessed in both patients and physicians. These variables explained 20.0% of the variance in patients' WTP (Nagelkerke’s R^2^ = 0.200), but only 15.2% of the WTP variance in physicians (Nagelkerke’s R^2^ = 0.152). In particular, higher income was a strong predictor of enhanced WTP in patients (β = 0.80, OR = 2.22, p<0.01) but less so in physicians (β = 0.59, OR = 1.80, p = 0.15). In physicians, living alone could be identified as a strong predictor for low WTP (= -1.39, OR = 0.25, p = 0.02). Models are summarized in Table 3.

**Table 3:** Multiple logistic regression model.

**Patients** (R^2^ = 0.200)	**β**	**SE**	**p**	**Odds Ratio (expβ)**	**95% CI**
Gender	-0.04	0.54	0.94	0.96	0.33–2.78
Age	-0.01	0.03	0.74	0.99	0.93–1.05
Income	0.80	0.30	<0.01	2.22	1.22–4.03
Living alone	-0.21	0.70	0.77	0.81	0.21–3.22
Employment	-0.35	0.82	0.67	0.71	0.14–3.54
Education level	0.09	0.18	0.60	1.10	0.77–1.57
Physicians* (R^2^ = 0.152)	**β**	**SE**	**p**	**Odds Ratio (expβ)**	**95% CI**
Gender	-0.76	0.60	0.21	0.47	0.14–1.53
Age	-0.03	0.05	0.55	0.97	0.88–1.07
Income	0.59	0.41	0.15	1.80	0.81–4.01
Living alone	-1.39	0.60	0.02	0.25	0.08–0.81

*Employment status and education level did not differ in physicians. Therefore, these variables were treated as fixed factors in this model.

In order to reduce relevant confounding factors in the comparison of patients’ and physicians’ WTP, we performed an ANCOVA using the group (patient vs. physician), gender, age and income as co-variates. In this way, we could show that group membership led to a significant result of the F-test for the questions concerning one-time payment. In case of continuous payments, income remained a strong predictive factor (Table 4).

**Table 4:** ANCOVA.

	One-time payment [k€]			One-time payment [% of income]			Continuous payment [€]			Continuous payment [% of income]		
	F	p	η^2^	F	p	η^2^	F	P	η^2^	F	p	η^2^
Corrected model	9.3	<0.01	0.39	3.3	<0.01	0.17	7.6	<0.01	0.34	2.5	<0.01	0.14
Intercept	242.3	<0.01	0.62	154.2	<0.01	0.49	323.2	<0.01	0.68	167.3	<0.01	0.51
Group	16,6	<0.01	0.10	6.6	0.01	0.04	9.3	<0.01	0.06	2.9	0.09	0.02
Gender	0.0	0.91	0.00	3.0	0.08	0.02	2.0	0.16	0.01	0.0	0.88	0.00
Age	1.7	0.15	0.04	1.5	0.20	0.04	1.0	0.39	0.03	0.7	0.58	0.02
Income	2.1	0.09	0.05	1.8	0.13	0.04	4.4	<0.01	0.10	2.9	0.02	0.07
R^2^	0.39			0.17			0.34			0.14		
R^2^ (adj.)	0.35			0.12			0.29			0.08		

## Discussion

This study was conducted in order to gain insight into WTP as an indicator for disease burden in patients with low-risk malignant melanoma. For ethical reasons, the study was conducted in patients with an at least 8 weeks preceding history of low-risk melanoma rather than in patients who are still involved in the primary tumor treatment process. To our knowledge, this is the first study evaluating willingness to pay directly as a total amount of money patients or physicians would pay to remain disease-free. Our patients had a much higher median WTP for one-time payment than patients with other chronic skin diseases. Patients with atopic dermatitis would pay a median sum of €1,000 (mean €11,884 ±80,690 range 0–1,000,000) [13], in patients with rosacea a median of €500 (mean €2,880 ± 16,784, range 0–300,000) was found [12], and patients with vitiligo reported a median WTP of €3,000 (mean €7,360 ± 41,970, range 0–1,000,000) [15]. A German study determined the value of ipilimumab with a two months survival prolongation but side effects [8]. Melanoma patients were willing to prefer ipilimumab over cash with 71% preferring therapy instead of money as opposed to 34% of healthy controls and 42% of physicians, respectively [8]. About two thirds of the patients preferred the therapy instead of the money for any sum (€10,000, €50,000€ and €100,000) in contrast to only 28% of healthy respondents and 43% of physicians who preferred ipilimumab over €100,000.

This does not match with a higher level of subjective health in our melanoma patients as a second indicator of disease burden. This disparity may be explained by the fact that patients with low-risk melanoma show little impairments in subjective health at the time of evaluation since they do not experience acute physical and emotional complaints. Nevertheless, there could be an overall feeling of anxiety of disease progression of the malignant melanoma that is not reflected in the EQ-VAS. The mere ideation of progressive disease may lead to a particular willingness to pay a relatively high amount of money just to get rid of any remaining risk.

As predictors of willingness to pay, age, income and employment status, subjective health status and anxiety were identified. In particular, elevated levels of emotional distress and gender are well-known predictors of WTP in other diseases [26, 27, 28]. When interpreting the WTP responses, it has to be considered that about 90% of Germans have statutory health insurance and thus do not receive bills or information about the actual cost of medical interventions. In a questionnaire based trial (n = 60, mainly breast cancer, also testicular, head and neck, lung, prostate) patients undergoing surveillance following curative therapy for localized cancer were questioned to rate three scenarios to elicit the maximum copayment they would be willing to pay for better treatment benefit [29]. Scenario A described a treatment for a curable cancer in terms of relapse risk. Scenarios B and C described therapies for irresectable cancer in terms of the 2-year survival chance and median life expectancy. Patients had a high WTP for more effective treatments (p < 0.05) [29]. In scenario B, patients who were employed demonstrated a greater willingness to pay (WTP) (odds ratio [OR], 12.6; 95% confidence interval [CI], 2.0–80.4), when controlling for efficacy. In scenario C, college graduates showed greater WTP and patients who reported previous financial burden showed lower WTP.

In our melanoma patient group, the quality of life is high measured by EQ-VAS and as well the patients showed high WTP. A point of criticism on the WTP concept is its dependence on sociodemographic factors, especially on the patients’ income. To minimize this problem, we modified the question by asking for the percentage of income in addition to absolute monetary values. We could confirm that income is an important factor in patients’ and physicians`WTP.

Further, WTP is principally subject to bias: The amount an individual is willing to pay in a theoretical situation might be unreasonable regarding the financial situation. In the current study, only a few participants indicated unrealistic sums (e. g. exceeding 100% of personal monthly income). A limitation of our study could also be the fact that up to 35.0% of patients dropped out. In comparison 25.9% of physicians did not fill out the questionnaire. These subjects were not evaluated in terms of sociodemographic factors. Therefore our results could be influenced by this bias.

Another remarkable result is the much higher WTP of physicians for sustainable cure from disease which is significant even after adjustment for gender, age and median income. Maybe the younger physician group is motivated by age and therefore is willing to invest more for a cure. The fact that physicians know very well how distant metastasis of melanoma can proceed may lead enhance WTP.

Another explanation might be the fact that physicians are completely aware of real drugs costs. When not asking about a cure but life prolongation under a tumor therapy WTP was lower in physicians. [8]. The higher WTP of physicians for cure may be due to the extensive knowledge about progressive melanoma disease while the lower WTP of physicians for life prolongation might be due to knowledge of side effects of tumor therapies. Why physicians are more willing to pay higher one time sums compared to continuous payments in comparison to patients, can only be speculated. One explanation could be a higher financial potency of the physicians in our study to receive bank credits. In contrast, continuous payments might be a higher burden for these young physicians. The characteristics of our physician cohort (younger, female) might be an explanation for the higher WTP. Why physicians living alone had a lower WTP can only be speculated. Possibly, these physicians had a lower financial strength or alternatively a lower subjective need to take care of their health because they don′t take care of a family. Therefore, it could be important to be aware of these influencing factors and take this into account in the decision making process with the patients. Physicians who have a higher WTP might be influenced to motivate their patients in a higher extent for adjuvant therapy. This stresses the need for standard guidelines and multidisciplinary discussion because the decision making process might be affected by the single opinion of the individual physicians.

The different WTP of patients and physicians should be taken into account as this disparity could influence the decision for a therapeutic approach. These differences between patients and their treating physicians could be key in this situation to vote for an adjuvant treatment or the avoidance of such a treatment due to undesired side effects.

In conclusion, our study indicates that low-risk melanoma patients have a high burden by their melanoma diagnosis. The identified differences with regard to WTP indicate that patients and physicians potentially rate melanoma disease and the risk of distant metastasis differentially. Therefore, this difference could influence the decision making process and should be taken into account.

## Supporting information

S1 Fig Original questionnaire (German). (DOCX)

S2 Fig Translated questionnaire (English). (DOCX)

## Abbreviations

EQ-5D: Quality of life questionnaire by EuroQol Group
EQ-VAS: Visual analogue scale within the EQ-5D
HADS: Hospital Anxiety and Depression Scale
HADS-A: Anxiety scale of HADS
HADS-D: Depression scale of HADS
irAEs: immune related adverse events
QoL: Quality of life
VAS: Visual analogue scale
WTP: Willingness to pay
