# Supplementary material for: Willingness to pay for a cure of low-risk melanoma patients in Germany
Source: PLoS One. 2018 May 24;13(5):e0197780. doi: 10.1371/journal.pone.0197780 (PMC5967822; doi:10.1371/journal.pone.0197780)
Supplement: S1 Fig — (DOCX) [file pone.0197780.s001.docx]

S1 Fig: Original questionnaire (German)
