# Supplementary material for: Willingness to pay for a cure of low-risk melanoma patients in Germany
Source: PLoS One. 2018 May 24;13(5):e0197780. doi: 10.1371/journal.pone.0197780 (PMC5967822; doi:10.1371/journal.pone.0197780)
Supplement: S2 Fig — (DOCX) [file pone.0197780.s002.docx]

S2 Fig: Translated questionnaire (English)

Willingness to pay (all values in Euro)

1.Given the fact, there would be a therapy that could prevent a melanoma relapse and this therapy would be a single tablet without side effects. How much money would you be willing to pay for this tablet as a one-time payment?

2. What percentage of your monthly net income are you willing to pay as a one-time payment for this treatment (if you are willing to pay more than your monthly income, then please indicate a value of more than 100%)?

3. Which total amount of money are you willing to pay continously every month for this treatment?

4. What percentage of your monthly net income are you willing to pay for this treatment continously every month (if you are willing to more than your monthly income, then please indicate a value of more than 100%)?
